# Mouse Models of Atherosclerosis: What They Teach Us, What They Miss, and When to Use Them

**DOI:** 10.3390/bioengineering13080911

**Published:** 2026-08-12

**Authors:** Xiaoxiao Geng, Annalisse R. McKee, Manuel Rosa-Garrido, Isidoro Cobo

**Affiliations:** 1 Division of Clinical Immunology & Rheumatology, Department of Medicine, Heersink School of Medicine, University of Alabama at Birmingham, Birmingham, AL 35294, USA; xgeng@uab.edu; 2 Department of Pathology, Heersink School of Medicine, University of Alabama at Birmingham, Birmingham, AL 35294, USA; mckeea@uab.edu; 3 Department of Cellular and Molecular Signaling, The Institute of Biomedicine and Biotechnology of Cantabria, CSIC-University of Cantabria, 39011 Santander, Spain; manuel.rosa@unican.es; 4 Department of Biomedical Engineering, School of Medicine, The University of Alabama at Birmingham, Birmingham, AL 35294, USA; 5 Comprehensive Arthritis, Musculoskeletal, Bone and Autoimmunity Center (CAMBAC), The University of Alabama at Birmingham, Birmingham, AL 35294, USA

**Keywords:** atherosclerosis, mouse models, lipoprotein metabolism, immunometabolism, vascular inflammation, plaque instability, single-cell RNA sequencing, coronary artery disease, model selection

## Abstract

Mouse models have been indispensable for defining the mechanisms of atherosclerosis, yet no single model reproduces the full spectrum of human disease. This review provides a practical framework for selecting mouse models according to the biological question being addressed. We compare classical lipoprotein-driven models, including *Apoe*^−/−^ and *Ldlr*^−/−^ mice, with models designed to study human-like lipoprotein metabolism, immune and immunometabolic mechanisms, adult-onset hypercholesterolemia, plaque complexity, coronary disease, and myocardial infarction-like outcomes. Particular emphasis is placed on how genetic background, lipoprotein composition, diet, sex, age, vascular site, and experimental design shape lesion development and influence interpretation. We also discuss discrepancies across single-cell RNA-sequencing studies, highlighting how differences in model, tissue processing, disease stage, and analytical strategy can alter the macrophage and immune-cell states detected. Rather than ranking models, we define what each model can teach, what it may miss, and when it is most appropriate to use. We conclude that future progress will depend on question-driven model selection, cross-model validation, improved reporting, and greater integration of molecular, spatial, and human benchmarking approaches.

## 1. Introduction

Atherosclerosis is the major pathological process underlying coronary artery disease, ischemic stroke, and peripheral vascular disease [1,2]. It develops over decades and is characterized by the progressive accumulation of lipids, inflammatory cells, smooth muscle cells, extracellular matrix, and necrotic debris within the arterial wall [3,4,5,6,7]. Although atherosclerosis was historically viewed primarily as a lipid-storage disorder, it is now recognized as a chronic immunometabolic disease in which dyslipidemia, endothelial dysfunction, innate and adaptive immune activation, vascular remodeling, and defective resolution of inflammation cooperate to drive lesion initiation, progression, and clinical complications [8,9,10,11,12].

Atherogenesis develops preferentially at arterial sites exposed to disturbed blood flow, such as branch points and curvatures [13]. These hemodynamic environments promote endothelial activation and facilitate the entry of apolipoprotein B-containing lipoproteins, including low-density lipoprotein (LDL), very-low-density lipoprotein (VLDL) remnants, and chylomicron remnants, into the subendothelial space [14]. Once retained within the intima, these lipoproteins undergo oxidative, enzymatic, and other chemical modifications that increase their inflammatory and immunogenic properties. Modified lipoproteins activate endothelial cells and vascular smooth muscle cells, inducing the expression of adhesion molecules and chemokines that recruit circulating leukocytes [15,16,17]. Monocytes subsequently infiltrate the developing lesion, differentiate into macrophages, and internalize modified lipoproteins through scavenger receptors, becoming lipid-laden foam cells. These early events establish a self-amplifying inflammatory circuit in which lipid accumulation and immune activation reinforce each other [9,11,18,19,20,21,22,23].

Vascular smooth muscle cells also play a central role in lesion development. They migrate from the media into the intima, proliferate, and produce extracellular matrix components that contribute to fibrous cap formation [19,20,21]. However, smooth muscle cells are not merely structural components of the plaque. Lineage-tracing and single-cell studies have demonstrated that they can acquire diverse phenotypic states, including macrophage-like and fibroblast-like identities, with potentially lesion-promoting or plaque-stabilizing functions [22,23,24]. Advanced atherosclerotic plaques therefore represent complex multicellular ecosystems in which plaque growth, stability, and the risk of rupture are determined by the balance among lipid deposition, inflammatory activation, matrix remodeling, cell death, and tissue repair [6,25].

Inflammation is no longer recognized as merely a secondary consequence of lipid accumulation but rather as a fundamental driver of atherosclerotic disease [26,27]. Human and experimental atherosclerotic lesions contain diverse immune cell populations, including macrophages, dendritic cells, T cells, B cells, and neutrophils. Monocyte recruitment is regulated by adhesion molecules such as selectins, VCAM-1, and ICAM-1, as well as by chemokine pathways including CCL2-CCR2, CX3CL1-CX3CR1 [28,29,30]. Within plaques, macrophages adopt heterogeneous activation states and produce inflammatory cytokines, proteases, and lipid mediators that influence lesion growth and stability [31,32]. Adaptive immunity also contributes substantially to disease pathogenesis. CD4^+^ T cells are abundant within atherosclerotic lesions with Th1 responses generally promoting disease progression, whereas regulatory T cells often exert protective effects, although both responses are highly context dependent [33,34,35]. Furthermore, evidence that T cells recognize ApoB-derived peptides supports the concept that atherosclerosis possesses an autoimmune component directed, at least in part, against modified or processed lipoprotein antigens.

Despite major advances in lipid-lowering therapies, substantial residual cardiovascular risk persists, and a significant proportion of this risk is inflammatory [36,37]. Clinical studies targeting inflammatory pathways have reinforced the concept that inflammation is not merely associated with atherosclerosis but contributes causally to cardiovascular events [38,39]. However, systemic immunosuppression carries significant risks, underscoring the need to identify disease-relevant mechanisms and develop experimental systems capable of disentangling the lipid-driven, immune-driven, metabolic, and vascular contributions to atherogenesis [39,40,41]. Consequently, experimental models remain indispensable tools for understanding disease mechanisms and for evaluating therapeutic strategies (Box 1).

Box 1 **Key differences between mouse and human atherosclerosis.**

**Lipoprotein metabolism.** In humans, most circulating cholesterol is transported in ApoB100-containing LDL particles, and additional factors such as CETP and lipoprotein(a) contribute to atherogenic lipoprotein metabolism. In contrast, wild-type mice transport most cholesterol in HDL, produce substantial amounts of ApoB48 through hepatic ApoB mRNA editing, lack CETP and lipoprotein(a), and are relatively resistant to spontaneous atherosclerosis.

**Plaque distribution and clinical complications.** Human atherosclerosis commonly affects the coronary, carotid, and peripheral arteries and may progress to plaque rupture or erosion, thrombosis, myocardial infarction, and stroke. Standard *Apoe*^−/−^ and *Ldlr*^−/−^ mice develop lesions predominantly in the aortic root, aortic arch, brachiocephalic artery, and proximal aorta, whereas spontaneous plaque rupture, occlusive coronary thrombosis, and myocardial infarction are uncommon.

**Disease development.** Human atherosclerosis develops over decades in the context of aging and the interaction of metabolic, environmental, and inflammatory risk factors. Murine atherosclerosis generally develops over weeks to months and usually requires genetic, viral, or dietary manipulation of lipoprotein metabolism. This accelerated induction facilitates mechanistic studies but may not reproduce the natural history of human disease.

**Immune and vascular responses.** Both mouse and human plaques exhibit endothelial activation, monocyte recruitment, macrophage foam-cell formation, adaptive immune responses, and smooth-muscle-cell phenotypic remodeling. However, the abundance, localization, and activation states of these populations vary between species, model, vascular site, diet, and disease stage.

**Translational implication.** No mouse model reproduces the complete spectrum of human atherosclerosis. Model selection should therefore be guided by the specific biological or clinical feature being studied, and major findings should, whenever possible, be validated in complementary models and benchmarked against human data.

Mouse models have been central to atherosclerosis research because they are relatively inexpensive, reproduce rapidly, are genetically tractable, and are highly amenable to dietary, pharmacological, lineage-tracing, and cell-specific genetic manipulations. However, mice do not naturally recapitulate human atherosclerosis [42,43]. Wild-type mice are relatively resistant to spontaneous lesion formation, in part because most circulating cholesterol is carried in high-density lipoproteins (HDL) rather than LDL. Moreover, lipoprotein metabolism, bile acid biology, vascular anatomy, and plaque progression differ substantially between mice and humans [43,44,45]. Consequently, robust experimental atherosclerosis in mice generally requires genetic or viral manipulation of lipid metabolism, often in combination with cholesterol-rich or high-fat diets.

The development of *Apoe*^−/−^ and *Ldlr*^−/−^ mice in the early 1990s transformed the field by providing reproducible and genetically tractable models of hypercholesterolemia-driven atherosclerosis [46,47]. These models remain the most widely used systems for studying lesion initiation, immune-cell recruitment, plaque progression, and candidate gene function. Nevertheless, they are not interchangeable. *Apoe*^−/−^ mice develop spontaneous hyperlipidemia and atherosclerosis even when maintained on a chow diet, largely because of impaired clearance of triglyceride-rich remnant lipoproteins. In contrast, *Ldlr*^−/−^ mice are more diet-dependent and predominantly accumulate ApoB100-containing LDL, particularly under cholesterol-rich dietary conditions [48,49,50,51]. Furthermore, ApoE and LDLR exert biological functions beyond plasma lipid regulation, influencing immune cell behavior, vascular cell function, and inflammatory responses. Consequently, findings obtained in one model cannot always be generalized to the other.

Over the past three decades, additional mouse models have been developed to address limitations of the classical *Apoe*^−/−^ and *Ldlr*^−/−^ systems. These include ApoE/LDLR double knockouts, ApoB100-only or *Ldlr*^−/−^/*Apobec*1^−/−^ models of LDL-driven familial hypercholesterolemia, humanized APOE3-Leiden and APOE3-Leiden.CETP mice, AAV-PCSK9 models that induce hypercholesterolemia without germline modification, somatic genome-editing approaches targeting *Ldlr*, and models designed to reproduce advanced plaque instability, coronary artery disease, myocardial infarction, or atherothrombosis [52,53,54,55,56,57,58,59]. Each model captures a distinct aspect of human disease while introducing its own biological constraints and experimental limitations.

Rather than providing a simple catalog of available models, this review examines how each model functions as a biological filter that shapes the interpretation of atherosclerosis mechanisms. We focus on how genetic design, lipoprotein context, diet dependence, immune perturbation, lesion distribution, and plaque complexity determine which questions a model can answer, and which conclusions it may inadvertently distort.

## 2. Models of Atherosclerosis

### 2.1. Lipoprotein-Driven Models of Lesion Initiation and Progression (Table 1)

The most widely used mouse models of atherosclerosis are those in which disease is driven primarily by perturbations in lipoprotein metabolism. This category includes the classical *Apoe*^−/−^ and *Ldlr*^−/−^ mice, as well as models developed to recapitulate specific aspects of human dyslipidemia, including *Ldlr*^−/−^/*Apobec1*^−/−^ mice, ApoB100-only models, human ApoB and ApoB/lipoprotein(a) transgenic mice, APOE3-Leiden and APOE3-Leiden.CETP mice, and more recent viral and somatic gene-editing approaches such as PCSK9-AAV, and AAV-CRISPR *Ldlr* models [53,54,55,60,61,62,63].

These models are essential because wild-type mice are naturally resistant to spontaneous atherosclerosis. Unlike humans, mice transport most circulating cholesterol in HDL-rich particles rather than LDL-rich particles and have very low levels of ApoB-containing atherogenic particles [43,50,64]. As a result, robust lesion formation generally requires experimental strategies (genetic, dietary, transgenic, viral, or somatic editing) that increase non-HDL cholesterol and promote retention of ApoB-containing lipoproteins in the arterial wall.

#### 2.1.1. Apoe^−/−^ Mice: The Classical Remnant-Driven Model

*Apoe*^−/−^ mice remain the most widely used and experimentally robust model of murine atherosclerosis [65]. APOE is a key ligand that mediates hepatic clearance of chylomicron and VLDL remnants through LDLR, LDL receptor-related protein 1, and heparan sulfate proteoglycans [66,67,68]. Loss of ApoE leads to the spontaneous accumulation of cholesterol-rich remnant lipoproteins and the development of hypercholesterolemia and atherosclerotic lesions, even in chow-fed animals with lesion formation accelerated by Western-type or high-fat/high-cholesterol diets [69,70]. The ease and reproducibility of lesion formation have made *Apoe*^−/−^ mice invaluable for studying lesion initiation, foam cell formation, vascular inflammation, immune-cell recruitment, candidate gene function, and therapeutic interventions including bone marrow transplantation and pharmacological intervention [71,72,73].

Importantly, *Apoe*^−/−^ mice should not be viewed simply as hyperlipidemic mice that develop atherosclerosis. ApoE has pleiotropic functions that extend beyond lipoprotein clearance. In addition to being produced by hepatocytes, *ApoE* is expressed by macrophages and other cell types, where it regulates cholesterol efflux, foam cell formation, oxidative stress, inflammatory signaling, immune regulation, smooth muscle cell behavior, and possibly platelet and endothelial cell function [74,75,76,77,78,79,80,81,82,83]. Consequently, phenotypes observed in *Apoe*^−/−^ mice reflect the combined effects of hyperlipidemia, plaque formation, and the systemic consequences of ApoE deficiency [66,84,85,86]. This consideration is particularly important when Apoe^−/−^ mice are used to study inflammatory or immunological pathways as findings may not necessarily translate to models where *ApoE* expression is preserved.

#### 2.1.2. Ldlr^−/−^ Mice: A Model of Diet-Induced LDL-Driven Disease

*Ldlr*^−/−^ mice provide a complementary model of atherosclerosis [43,46]. In contrast to *Apoe*^−/−^ mice, chow-fed *Ldlr*^−/−^ mice develop little spontaneous atherosclerosis and generally require cholesterol-containing or Western-type diets to induce robust lesion formation [50,87,88]. Under these conditions, this mouse model accumulates LDL and VLDL particles and develops lesions in the aortic root, aortic arch, brachiocephalic artery, and *en face* aorta. Because ApoE expression remains intact, *Ldlr*^−/−^ mice are often considered more representative of human LDL/ApoB-driven disease [50]. Their dependence on dietary manipulation is both an advantage and a limitation. On one hand, it allows investigators to tune the onset, duration, and severity of hypercholesterolemia. On the other, it makes experimental outcomes highly sensitive to diet composition, cholesterol content, fat source, cholate, duration of feeding, sex, microbiota, and genetic background. The distinction between *Apoe*^−/−^ and *Ldlr*^−/−^ mice is therefore not merely technical. *Apoe*^−/−^ mice predominantly accumulate cholesterol-rich ApoB48-containing remnant particles, whereas *Ldlr*^−/−^ mice, particularly under specific diet conditions, more closely model accumulation of ApoB100-containing LDL. As a result, interventions that produce similar effects in one model may behave differently in the other depending on lipoprotein context, diet, lesion stage, or vascular bed [50].

#### 2.1.3. Distinct Immune Landscapes in Apoe^−/−^ and Ldlr^−/−^ Mice

Single-cell studies further demonstrate that *Apoe*^−/−^ and *Ldlr*^−/−^ mice are not immunologically equivalent. Although a high-fat diet promotes myeloid expansion in both models, the composition of the immune response differs substantially by genotype: *Apoe*^−/−^ aortas are enriched in B-cell populations, including B2 cells and a small B1-like cluster, whereas *Ldlr*^−/−^ lesions contain a larger macrophage representation. Within the macrophage compartment, scRNA-seq identifies resident-like macrophages, inflammatory macrophages enriched for *Il1b*, *Ccl2*, *Ccl3*, *Ccl4*, *Cxcl2*, *Tnf*, and *Cd14*, IFN-inducible macrophages, and *TREM2*^hi^ foamy macrophages enriched for *Trem2*, *Cd9*, *Lgals3*, *Ctsd*, *Fabp5*, and lipid/catabolic programs [8,89,90]. These differences have important experimental implications. An intervention that appears to alter plaque inflammation may be acting on distinct biological processes depending on the model used, including B-cell accumulation, macrophage expansion, inflammatory macrophage activation, IFN-inducible programs, or lipid-handling *TREM2*^hi^ foam-cell biology. Thus, similar phenotypic outcomes may arise through fundamentally different immune mechanisms.

#### 2.1.4. The Influence of Genetic Background

Genetic background further modifies atherosclerotic susceptibility, lesion composition, and plaque distribution. Although C57BL/6 mice are widely used because of their susceptibility to atherosclerosis, other backgrounds, such as C3H/HeJ, FVB/N, and BALB/c, are comparatively resistant to disease [57,91,92,93]. Studies using *Apoe*^−/−^ mice on FVB/N, BALB/c, 129/SvEv, and DBA/2J backgrounds have demonstrated marked strain-dependent differences in lesion burden, inflammatory responses, and vascular-site susceptibility [94,95,96,97]. For example, FVB/N-*Apoe*^−/−^ mice develop substantially smaller aortic-root lesions than C57BL/6-*Apoe*^−/−^ mice despite higher total cholesterol, whereas 129/SvEv-*Apoe*^−/−^ mice show delayed lesion initiation in the aortic root but earlier and more extensive disease in the aortic arch, highlighting the contribution of vascular anatomy and local hemodynamics [94,96]. Similar background-dependent effects have been observed in *Ldlr*^−/−^ mice, with C57BL/6 and FVB/N strains displaying different lesion susceptibility under comparable dietary conditions [98]. Interestingly, a recent comparison of *Apoe*^−/−^ and *Ldlr*^−/−^ mice on the atherosclerosis-resistant C3H background showed that high-fat diet-fed C3H-*Ldlr*^−/−^ mice developed larger aortic-root lesions than C3H-*Apoe*^−/−^ mice, despite lower MCP-1 levels and similar oxidative-stress markers [70]. These findings suggest that lipoprotein characteristics, including the abundance of small, dense LDL particles, may under some conditions be more important determinants of lesion formation than systemic inflammatory markers. Collectively, these studies underscore that the relative severity of *Apoe*^−/−^ and *Ldlr*^−/−^ models depends on interactions among the disrupted gene, genetic background, diet, sex, and vascular site.

#### 2.1.5. Models of Human LDL-Rich Familial Hypercholesterolemia

Several models have been developed to more closely reproduce human LDL-rich familial hypercholesterolemia [99,100,101]. A major species difference is that mouse liver extensively edits ApoB mRNA, favoring ApoB48 production, whereas human liver produces ApoB100 [102,103]. Consequently, standard *Ldlr*^−/−^ mice do not fully recapitulate human LDL-C-driven familial hypercholesterolemia. *Ldlr*^−/−^/Apobec1^−/−^ mice address this limitation by combining LDLR deficiency with loss of ApoB mRNA editing, thereby forcing production of ApoB100 [53,104,105]. These mice develop markedly elevated LDL cholesterol and ApoB100 levels, along with extensive atherosclerosis, even on a low-fat chow diet [53,106]. Similarly, ApoB100-only models and related human ApoB models emphasize the role of ApoB100-containing particles in lesion formation and are particularly useful for studying LDL particle biology, ApoB100-mediated retention, familial hypercholesterolemia, and LDL-targeted therapeutic intervention. Their main limitation is practical: they require more complex breeding strategies and are less amenable to rapid genetic crosses than *Apoe*^−/−^ or *Ldlr*^−/−^ [49].

#### 2.1.6. Human ApoB and Lipoprotein(a) Transgenic Models

Human ApoB-transgenic mice provide a complementary strategy for modelling human-like ApoB-containing lipoproteins while preserving endogenous murine clearance pathways [107,108,109,110]. High-expressing lines produce human ApoB100 predominantly in LDL-sized particles and can develop increased VLDL/LDL cholesterol and extensive aortic atherosclerosis, particularly following dietary challenge [88]. These models are therefore useful for studying ApoB production, LDL-particle clearance and retention, lipoprotein oxidation, lipid-driven vascular inflammation, and interventions directed against LDL- or ApoB-containing particles. Their principal limitation is that human ApoB expression alone may produce modest or variable hypercholesterolemia, depending on the transgenic line, and robust lesion development frequently requires an atherogenic diet or combination with additional genetic modifications. Moreover, the presence of human ApoB does not correct other species differences in lipoprotein metabolism, vascular anatomy, or plaque progression. Seminal studies of human ApoB-transgenic mice directly demonstrated the formation of human-like LDL particles and increased aortic atherosclerosis in high-expressing lines.

Models producing human lipoprotein(a) are even more specialized. Mice do not naturally express apo(a), and human apo(a) associates inefficiently with endogenous mouse ApoB [43]. Consequently, authentic human-like Lp(a) particles are generally generated by combining human apo(a) and human ApoB transgenes. These models enable direct investigation of ApoB100–apo(a) assembly, the proatherogenic effects of Lp(a), oxidized phospholipid biology, and therapeutic approaches designed to reduce ApoB or apo(a) expression. However, circulating Lp(a) concentrations and apo(a) isoform characteristics depend strongly on transgene design and expression level and may not reproduce the lifelong exposure or broad apo(a) size heterogeneity observed in humans. In addition, these mice rarely reproduce advanced human complications such as spontaneous plaque rupture, coronary thrombosis, or myocardial infarction. Human ApoB/apo(a) double-transgenic mice have nevertheless provided experimental evidence that Lp(a) is more atherogenic than free apo(a), and they have been used to test antisense-based lipid-lowering strategies.

#### 2.1.7. APOE3-Leiden and APOE3-Leiden.CETP Mice

APOE3-Leiden mice represent another important class of lipoprotein-driven models. The *APOE3*-Leiden mutation causes a dominant form of familial dysbetalipoproteinemia in humans [111]. Transgenic mice carrying the human APOE3-Leiden construct develop diet-responsive hyperlipoproteinemia characterized by marked increases in cholesterol and triglycerides, primarily within VLDL/LDL-sized fractions [112,113]. APOE3-Leiden mice develop lesions ranging from macrophage-rich fatty streaks to advanced plaques containing oxidized lipoprotein epitopes, smooth muscle cell cap formation, fibrosis, cholesterol crystals, and necrotic features [43,114,115]. Because these mice preserve endogenous mouse ApoE expression in macrophages and other tissues, they provide an alternative to complete ApoE deficiency for studying remnant lipoprotein metabolism and diet-responsive dysbetalipoproteinemia-like atherosclerosis.

APOE3-Leiden.CETP mice extend this approach by introducing human cholesteryl ester transfer protein (CETP), a key component of human lipoprotein metabolism that is absent in mice. CETP expression shifts cholesterol distribution from HDL toward VLDL/LDL, reduces plasma-mediated SR-BI-dependent cholesterol efflux, and markedly increases atherosclerotic lesion area in APOE3-Leiden mice [116]. Because APOE3-Leiden.CETP mice exhibit a more human-like lipoprotein profile and retain an intact ApoE–LDLR remnant-clearance pathway, they are particularly useful for testing lipid-modifying therapies, including interventions targeting VLDL/LDL metabolism, HDL biology, CETP function, and remnant clearance [117,118,119]. Their main limitations are that they are more diet-dependent, develop atherosclerosis more slowly, and are less genetically flexible and less amenable to mechanistic crosses than *Apoe*^−/−^ or *Ldlr*^−/−^ mice.

The predictable and adjustable dietary response of APOE3-Leiden.CETP mice makes them particularly valuable for pharmacological studies of dysbetalipoproteinemia, mixed dyslipidemia, remnant metabolism, and lipid-modifying therapies. Nevertheless, the introduction of human APOE3-Leiden and CETP does not overcome the murine vascular anatomy and immune context, and spontaneous plaque rupture, coronary thrombosis, and myocardial infarction remain uncommon. In addition, transgenic expression of APOE3-Leiden and CETP represents a defined experimental perturbation rather than the broader genetic and metabolic heterogeneity of human dyslipidemia.

#### 2.1.8. Viral and Somatic Gene-Editing Approaches

Viral and somatic gene-editing approaches have expanded the lipoprotein-driven toolkit by eliminating the need for extensive breeding strategies. AAV-mediated expression of gain-of-function PCSK9 promotes hepatic LDLR degradation, producing sustained hypercholesterolemia and atherosclerosis after a single injection, usually combined with Western-type or high-fat diet feeding [120,121]. This approach enables rapid induction of atherosclerosis in virtually any genetic background, including conditional knockouts, reporter strains and aged mice [122]. However, PCSK9-AAV should not be considered an interchangeable substitute for *Ldlr*^−/−^ mice. PCSK9 is expressed at supraphysiological levels and may exert effects beyond LDLR degradation, particularly in studies involving lipoprotein metabolism or macrophage biology [123,124,125].

AAV-CRISPR *Ldlr* models provide a related but mechanistically distinct approach by directly disrupting hepatic *Ldlr* in adult mice [55]. These models induce severe hypercholesterolemia and atherosclerosis with a single injection, avoiding the need for germline *Ldlr*^−/−^ breeding. Importantly, direct comparison between AAV-CRISPR *Ldlr* and AAV-hPCSK9 showed that both approaches generate atherosclerosis, but their efficiency can differ by sex, with AAV-CRISPR performing better for *Ldlr* removal in male mice and AAV-hPCSK9 being more effective in female mice [55]. Somatic editing also enables genetic-interaction studies [126,127]. For example, hepatic disruption of *Ldlr* induces hypercholesterolemia and atherosclerosis, whereas concomitant disruption of *Apob* prevents disease by reducing hepatic ApoB production [128,129]. These features make AAV-CRISPR models especially useful for adult-onset metabolic disease modeling, therapeutic target validation, and rapid testing of candidate genes. Nevertheless, they are subject to limitations common to somatic editing approaches, including variable editing efficiency, incomplete deletion, potential off-target effects, vector-related confounders, and the need for proper controls.

#### 2.1.9. Summary of Lipoprotein-Driven Models of Lesion Initiation and Progression Models

Collectively, lipoprotein-driven models form the experimental foundation of murine atherosclerosis research, but each represents a distinct biological context rather than a generic model of disease. *Apoe*^−/−^ mice emphasize remnant-driven hyperlipidemia and complete loss of ApoE biology; *Ldlr*^−/−^ mice recapitulate diet-dependent LDL/ApoB-driven disease with preserved ApoE; *Ldlr*^−/−^/*Apobec1*^−/−^ and ApoB100-only models closely reproduce human-like LDL-C and ApoB100 excess; human ApoB and Lp(a) transgenic models isolate specific human lipoprotein particles; APOE3-Leiden and APOE3-Leiden.CETP mice capture diet-responsive remnant metabolism and human-like responses to lipid-modifying therapies; and PCSK9-AAV or AAV-CRISPR models offer rapid, flexible, and adult-onset induction of hypercholesterolemia. Therefore, model selection should begin with the underlying lipoprotein question: Is the study focused on remnant lipoproteins, LDL/ApoB100, human ApoB production, Lp(a), humanized lipid pharmacology, rapid disease induction, or the interaction between dyslipidemia and a second biological process?

In addition to selecting the appropriate lipoprotein context, investigators must consider the time required to reach the plaque stage relevant to the biological question. Representative timelines for the development of early and more complex lesions in commonly used *Apoe*^−/−^, *Ldlr*^−/−^, and PCSK9-AAV models are summarized in Table 2. These timelines should be regarded as practical starting points rather than fixed thresholds because lesion progression is influenced by sex, age, genetic background, diet composition, vascular site, and experimental endpoint.

### 2.2. Immunoinflammatory and Immunometabolic Mechanism Models (Table 1)

Atherosclerosis is an inflammatory and immunometabolic disease, but there is no commonly used mouse model in which atherosclerosis is driven by immunity alone. Robust murine lesion formation almost always requires a hyperlipidemic background. Therefore, “immunoinflammatory models” should be understood as lipid-driven models of atherosclerosis that are particularly useful for interrogating immune, inflammatory, or immunometabolic mechanisms. This distinction is important. The goal is not to identify a purely immune model but rather to select a lipid context in which immune mechanisms can be interpreted clearly.

#### 2.2.1. *Ldlr*^−/−^ Mice for Immune and Inflammatory Studies

*Ldlr*^−/−^ mice are often the most appropriate classical model for immune and inflammatory studies. Because ApoE expression remains intact, immune phenotypes are less likely to be confounded by complete loss of ApoE-dependent macrophage and immune regulation [43,75,86]. *Ldlr*^−/−^ mice are therefore well suited for studies of monocyte production, macrophage recruitment, cytokine signaling, inflammasome activation, T-cell responses, sterile inflammatory triggers, trained immunity, bone marrow transplantation, and cell-specific gene deletion [43,130,131,132]. Their diet dependence can also be advantageous experimentally. Short-term high-fat diet feeding can be used to study early leukocyte recruitment and foam-cell formation, whereas prolonged feeding enables the investigation of lesion progression, necrotic core formation, and plaque composition. However, this same diet dependence introduces an important interpretative challenge. Differences in cholesterol content, fat source, carbohydrate composition, cholate, microbiota, sex, age, and feeding duration can substantially alter systemic metabolism and immune tone [133,134].

#### 2.2.2. Apoe^−/−^ Mice and the Challenge of ApoE Deficiency

*Apoe*^−/−^ mice remain highly valuable for immunological studies because much of the atherosclerosis literature has been generated using this background, including studies of monocyte recruitment, macrophage activation, chemokines, adhesion molecules, T-cell polarization, B-cell responses, cytokine signaling, and innate immune activation [135,136,137,138]. The robustness of lesion formation also makes *Apoe*^−/−^ mice attractive when substantial disease burden is required without prolonged diet intervention. However, immune studies in *Apoe*^−/−^ mice require more cautious interpretation. ApoE is not merely a lipoprotein ligand; it also regulates macrophage cholesterol efflux, inflammatory activation, oxidative stress, antigen presentation, T-cell responses, endothelial activation, and smooth muscle cell behavior [139,140,141]. Consequently, immune phenotypes observed in *Apoe*^−/−^ mice may reflect interactions among the pathway under investigation, hyperlipidemia, and the absence of ApoE itself. For this reason, immune mechanisms identified in *Apoe*^−/−^ mice are most compelling when validated in *Ldlr*^−/−^, PCSK9-AAV, or other *ApoE*-intact models.

#### 2.2.3. Comparing Apoe^−/−^ and Ldlr^−/−^ Backgrounds

The comparison between *Apoe*^−/−^ and *Ldlr*^−/−^ mice is particularly informative for immunoinflammatory studies. Some interventions produce similar effects in both backgrounds, supporting broad relevance to atherosclerosis [50]. Others differ between models, indicating that the pathway depends on lipid context, ApoE biology, diet, lesion stage, or vascular site. Such discordance should not be dismissed as experimental noise. Instead, it may reveal whether a mechanism is remnant-dependent, LDL-dependent, ApoE-dependent, diet-dependent, or stage-specific. For example, studies in genetically resistant C3H mice showed that high-fat diet-fed *Ldlr*^−/−^ mice developed larger lesions than *Apoe*^−/−^ mice despite lower systemic MCP-1 levels, suggesting that lipoprotein quality, including the abundance of small dense LDL particles, may outweigh some systemic inflammatory markers in determining lesion susceptibility [70,91]. These observations reinforce the broader concept that immune pathways in atherosclerosis cannot be interpreted independently of the lipoprotein environment in which they are studied.

#### 2.2.4. Adult-Onset PCSK9-AAV Models

PCSK9-AAV models (AAV8, commonly 1–5 × 10^11^ vg/mouse, administered intravenously, typically by tail-vein injection) are especially useful for immunoinflammatory and immunometabolic studies because they allow adult-onset atherosclerosis to be induced in existing strains without germline disruption of *Apoe* or *Ldlr*. This feature is particularly valuable when investigators already have conditional knockout strains, Cre lines, reporter mice, aged cohorts, or disease-prone colonies. For example, PCSK9-AAV can be used to induce hypercholesterolemia in young and aged mice at defined time points, reducing the confounding effect of lifelong *Apoe*^−/−^ or *Ldlr*^−/−^ deficiency, in which atherosclerosis and aging progress simultaneously [142]. It can also be applied to complex genetic backgrounds where breeding into *Apoe*^−/−^ or *Ldlr*^−/−^ backgrounds would be costly and time consuming. Consequently, PCSK9-AAV models are attractive for studies of macrophage biology, immunometabolism, aging, trained immunity, clonal hematopoiesis, sterile inflammation, and systemic inflammatory disease. Although initially established primarily in male C57BL/6 mice, the AAV-PCSK9 model has been successfully applied in female mice and multiple mouse strains, where it consistently induces PCSK9 expression, LDL receptor degradation, hypercholesterolemia, and atherosclerotic lesion formation. Therefore, sex- and strain-dependent differences in lipid metabolism, immune responses, and plaque characteristics may influence disease severity and should be considered when designing experiments and interpreting translational relevance.

#### 2.2.5. AAV-CRISPR Ldlr Models

The AAV-PCSK9 model becomes particularly relevant to immunometabolism when combined with a high-fat, high-cholesterol diet. Under these conditions, AAV-PCSK9-injected C57BL/6 mice develop atherosclerosis together with metabolic dysfunction, including insulin resistance, impaired glucose tolerance, pancreatic islet hyperplasia, and increased pro-inflammatory Ly6C^high/med^ monocytes in adipose tissue [143]. These features overlap with those observed in HFD-C-fed *Ldlr*^−/−^ mice and make the model useful for studying the intersection of hypercholesterolemia, obesity-associated inflammation, glucose dysregulation, adipose tissue immune remodeling, and atherosclerosis [143]. Importantly, these metabolic phenotypes cannot necessarily be explained solely by elevated plasma cholesterol. Diet composition and systemic metabolic stress may independently shape immune tone and lesion biology. Therefore, AAV-PCSK9 combined with HFD-C should be viewed as an integrated metabolic syndrome/atherosclerosis model rather than simply a faster version of *Ldlr*^−/−^ mice.

AAV-CRISPR *Ldlr* models provide another flexible platform for immune and immunometabolic studies. By producing somatic disruption of hepatic *Ldlr* in adult mice, this approach enables disease induction in existing genetic backgrounds and avoids long breeding schemes [55]. It may be especially useful for studying genes with developmental effects, genes requiring multiple alleles, or candidate pathways identified through genetic screens. Somatic editing can also be combined with the disruption of other hepatic genes, as demonstrated by co-targeting *Ldlr* and *ApoB* to show that reducing hepatic ApoB production prevents hypercholesterolemia and atherosclerosis [144,145]. However, studies using AAV-CRISPR must account for vector exposure, editing efficiency, possible off-target effects, sex differences, and the distinction between adult-onset somatic deletion and lifelong germline deficiency.

#### 2.2.6. Metabolic Syndrome and Immunometabolic Disease Models

Models of obesity, diabetes, aging, and metabolic syndrome also belong within the immunometabolic category. Atherosclerosis in humans rarely develops in isolation; instead, it commonly occurs in the setting of obesity, insulin resistance, diabetes, hypertension, chronic kidney disease, clonal hematopoiesis, or recurrent systemic inflammation [146,147,148,149,150,151]. *Ldlr*^−/−^ mice fed high-fat (HFD) or HFD-C diets, *Ldlr*^−/−^ mice crossed with ob/ob or db/db models, *Apoe*^−/−^ mice exposed to diabetic or hypertensive stimuli, and PCSK9-AAV mice fed HFD-C can all be used to examine how metabolic stress reshapes atherogenesis [46]. These models are useful for studying monocyte production, adipose inflammation, glucose-lipid interactions, endothelial dysfunction, macrophage polarization, trained immunity, and systemic inflammatory priming. Their main limitation is interpretive complexity. When lesion size changes, the underlying mechanism may involve alterations in plasma lipids, body weight, glucose metabolism, insulin signaling, adipokines, blood pressure, the microbiome, immune activation, or vascular responsiveness. Consequently, these models are powerful for studying integrated disease biology but are less suitable for isolating individual immune pathways.

#### 2.2.7. Bone Marrow Transplantation and Conditional Genetics

Bone marrow transplantation and conditional genetic approaches are central tools within this category. Historically, bone marrow chimeras in *Apoe*^−/−^ or *Ldlr*^−/−^ recipients were used to distinguish hematopoietic from non-hematopoietic contributions to lesion development [152,153]. More recently, cell-specific Cre-lox approaches, inducible gene deletion, fate mapping, and single-cell transcriptomic studies have increased the precision with which macrophages, dendritic cells, T cells, B cells, endothelial cells, smooth muscle cells, and other populations can be studied. For these approaches, model choice becomes especially important. If the question concerns macrophage-intrinsic immune regulation, *Ldlr*^−/−^ or PCSK9-AAV models in a floxed background may be preferable because *ApoE* expression is preserved. If the goal is to compare findings directly with decades of published immune studies, *Apoe*^−/−^ may be appropriate, but conclusions should be interpreted within the context of *ApoE* deficiency.

#### 2.2.8. Macrophage Heterogeneity and Model Interpretation

Single-cell and fate-mapping studies are particularly relevant for immune-focused models because they demonstrate that macrophages do not represent a single inflammatory phenotype. Lesional macrophages include inflammatory macrophages enriched for *Il1b*, *Ccl2*, *Ccl3*, *Ccl4*, *Cxcl2*, *Tnf*, *Cd14*, and *Nlrp3*; IFN-inducible macrophages characterized by interferon-stimulated genes; TREM2^hi^ foamy macrophages associated with lipid handling, lysosomal catabolism, and plaque remodeling; and resident-like macrophages that partially overlap with vascular or adventitial macrophage programs [8,90]. In addition, MacAIR (aortic intima resident macrophages) contribute to the earliest foam cell populations, whereas monocyte-derived macrophages progressively dominate as plaques progress [89,154,155]. Consequently, immune perturbation models should be interpreted according to the cellular process being investigated, including cytokine signaling, IFN activation, monocyte recruitment, resident macrophage biology, foam-cell formation, or regression-associated remodeling. This consideration is particularly important in studies using bone marrow chimeras, myeloid Cre drivers, macrophage depletion, or cytokine blockade, where the same intervention may affect distinct macrophage origins or activation states.

#### 2.2.9. Summary of Immunoinflammatory and Immunometabolic Mechanism Models

The central message of this section is that immune studies require a model matched to the biological question being asked. *Apoe*^−/−^ mice are robust and historically important but are immunologically confounded by the absence of ApoE. *Ldlr*^−/−^ mice develop less spontaneous disease but often provide a cleaner background for studying immune mechanisms. PCSK9-AAV and AAV-CRISPR *Ldlr* models are flexible and particularly useful for complex genetic backgrounds, aging, and immunometabolism, although they introduce additional considerations related to viral delivery, somatic editing, sex-dependent effects, and adult-onset disease induction. Models of metabolic syndrome increase translational relevance but sacrifice mechanistic simplicity. Therefore, the optimal model depends on whether the objective is to isolate a cell-intrinsic immune mechanism, model systemic immunometabolic disease, test a candidate inflammatory pathway in established plaques, or reproduce a clinically relevant metabolic comorbidity.

## 3. Models of Plaque Complexity, Coronary Disease, and Clinical-Event-like Phenotypes (Table 1)

### 3.1. Why Advanced Plaque Models Are Needed

The third category includes models designed to capture advanced features of human atherosclerosis that are poorly reproduced by standard *Apoe*^−/−^ and *Ldlr*^−/−^ mice. This category is particularly important because the clinical consequences of human atherosclerosis arise not simply from the presence of plaques but from plaque growth, luminal obstruction, plaque rupture or erosion, thrombosis, myocardial infarction, stroke, and heart failure. Classical mouse models are highly useful for studying lesion formation in the aortic root, aortic arch, brachiocephalic artery, and *en face* aorta, but they rarely develop spontaneous plaque rupture, occlusive coronary thrombosis, myocardial infarction, or sudden death. As a result, a model that is excellent for measuring aortic lesion burden may be inadequate for studying plaque instability or coronary events.

### 3.2. Limitations of Classical Apoe^−/−^ and Ldlr^−/−^ Models

*Apoe*^−/−^ and *Ldlr*^−/−^ mice can develop complex aortic lesions characterized by lipid cores, macrophage accumulation, smooth muscle cell involvement, and fibrous cap-like structures, particularly after prolonged dietary challenge or additional genetic and environmental stressors [43,46,70,156]. However, spontaneous coronary artery occlusion and myocardial infarction remain uncommon. Lesions predominantly develop in the aortic root and large proximal arteries rather than in the coronary circulation. This anatomical mismatch represents a major translational limitation for studies of myocardial infarction, ischemia–reperfusion injury, plaque rupture, and cardioprotective therapies. Therefore, when the biological question concerns clinical events rather than lesion burden, more specialized models are required.

### 3.3. Apoe^−/−^ Fbn1C1039G^+/−^ Mice: Plaque Vulnerability and Rupture-Prone Disease

One approach is to modify vessel wall integrity to promote plaque vulnerability, as in *Apoe*^−/−^ Fbn1C1039G^+/−^ mice. Mutation of fibrillin-1 alters extracellular matrix structure, and in the setting of *Apoe* deficiency, promotes features of advanced plaque pathology that more closely resemble human vulnerable plaques, including larger necrotic cores, intraplaque neovascularization, hemorrhage, plaque rupture-like events, thrombosis, myocardial infarction, stroke, and in some reports, sudden death [157]. This model is particularly useful for studying plaque-stabilizing therapies, matrix remodeling, intraplaque hemorrhage, and mechanisms linking plaque structure to thrombotic complications. Its main limitation is that it deliberately introduces an extracellular matrix defect and therefore may not accurately reflect the natural progression of human atherosclerosis. It is best viewed as a model of plaque vulnerability and rupture-prone disease rather than a general model of atherogenesis.

### 3.4. Angiotensin II Models: Vascular Stress and Plaque Destabilization

Angiotensin II infusion in *Apoe*^−/−^ mice represents another strategy to increase vascular inflammation, hypertension-related stress, aneurysm formation, and features of plaque destabilization [158,159,160]. This model is useful for studying interactions among blood pressure, vascular inflammation, aneurysm biology, medial remodeling, and plaque vulnerability. However, it is less suitable as a model of spontaneous human plaque rupture because AngII introduces a potent systemic stimulus that simultaneously affects blood pressure, inflammatory signaling, aneurysm formation, and vascular remodeling [159,161]. Consequently, AngII-based models are best reserved for questions that explicitly involve hemodynamic or inflammatory vascular stress, rather than as a generic method for generating more severe atherosclerosis.

### 3.5. SR-BI^−/−^/Apoe^−/−^ Mice: Coronary Disease and MI-like Complications

SR-BI-based models are among the few murine models that reproducibly develop coronary atherosclerosis and myocardial infarction-like complications. SR-BI is a major HDL receptor that mediates the selective uptake of HDL cholesteryl esters by the liver and steroidogenic tissues. Loss of SR-BI disrupts HDL metabolism and reverse cholesterol transport. When SR-BI deficiency is combined with Apoe deficiency, mice develop severe dyslipidemia, abnormal lipoprotein particles, occlusive coronary artery lesions, spontaneous myocardial infarctions, cardiac dysfunction, electrocardiographic abnormalities, cardiac enlargement, reduced ejection fraction, and premature death. These features make SR-BI^−/−^/*Apoe*^−/−^ mice unusually valuable for studying coronary atherosclerosis and MI-like complications in a murine system [162,163]. Their main limitation is disease severity. The phenotype develops rapidly and is accompanied by abnormal lipoprotein morphology and systemic pathology that differ substantially from typical human atherosclerosis. Thus, this model is highly useful for studying coronary-event biology but less suitable for investigating slow, multifactorial plaque progression.

### 3.6. SR-BI^−/−^/ApoeR61h/h Mice: Diet-Inducible Coronary Disease

SR-BI^−/−^/ApoeR61h/h mice were developed to provide greater control over the timing of coronary disease. ApoeR61h/h mice express reduced levels of a hypomorphic *APOE* variant. When combined with SR-BI deficiency, these mice remain relatively protected on chow diet but develop diet-induced hypercholesterolemia, occlusive coronary atherosclerosis, myocardial infarction, cardiac dysfunction, and premature death when challenged with an atherogenic diet [162,164]. This diet-inducible behavior is a major advantage because investigators can control disease onset, severity, duration, and potentially regression. The model is particularly useful for testing therapies directed at advanced coronary lesions, cardiac dysfunction, or diet-induced progression. However, as with other SR-BI models, the phenotype is strongly influenced by major disturbances in HDL metabolism and lipoprotein handling and should therefore be regarded as a specialized model of coronary disease rather than a universal model of atherosclerosis.

### 3.7. SR-BI^−/−^/Ldlr^−/−^ Mice: Coronary Pathology with Preserved ApoE Expression

SR-BI^−/−^/*Ldlr*^−/−^ mice provide another model of coronary disease, particularly under atherogenic diet conditions [165,166,167]. Because ApoE expression is preserved, this model may avoid some of the confounding effects associated with ApoE deficiency while still generating advanced coronary lesions and myocardial pathology in certain settings. It may therefore be particularly relevant for studies examining the interaction between LDLR deficiency and impaired HDL/SR-BI function. However, the combination of defective selective cholesterol uptake, elevated HDL, LDLR deficiency, and severe dyslipidemia creates a highly complex lipoprotein environment. As with SR-BI^−/−^/Apoe^−/−^ mice, the model’s strength is its ability to generate coronary pathology, albeit at the cost of metabolic complexity.

### 3.8. Apoe^−/−^/Ldlr^−/−^ Mice: Severe Hyperlipidemic Atherosclerosis

*Apoe*^−/−^/*Ldlr*^−/−^ double knockout mice can develop more severe hyperlipidemia and more advanced lesions than either single knockout strain alone. Following prolonged high-fat, high-cholesterol feeding, these mice may also develop coronary involvement [57,168]. They are useful when a substantial lipid-driven disease burden is required and can serve as an intermediate model between classical lesion models and more aggressive coronary disease models. However, they do not consistently reproduce spontaneous plaque rupture or myocardial infarction to the extent seen in SR-BI-based models. Their principal value lies in modeling severe hyperlipidemic atherosclerosis rather than clinical cardiovascular events.

### 3.9. Modeling Myocardial Infarction and Ischemia–Reperfusion Injury

For studies of myocardial infarction and ischemia–reperfusion injury, model choice becomes especially important. Most experimental myocardial infarction (MI) studies rely on left anterior descending (LAD) coronary artery ligation or ischemia–reperfusion protocols in young, otherwise healthy mice, whereas human MI usually occurs in older individuals with coronary atherosclerosis, vascular dysfunction, metabolic comorbidities, and plaque rupture or erosion [169,170,171,172,173]. Standard *Apoe*^−/−^ and *Ldlr*^−/−^ mice generally fail to develop substantial coronary plaque burden and therefore may not adequately reproduce the vascular context in which human MI occurs. Specialized models with coronary lesions, including SR-BI-based models and potentially *Apoe*^−/−^ Fbn1C1039G^+/−^ mice, may provide more relevant platforms for investigating how atherosclerosis influences coronary function, infarct size, reperfusion injury, and cardioprotective therapies. Nevertheless, these models also introduce severe and, in some cases, nonphysiological disturbances in lipid metabolism. Experimental design must therefore balance clinical relevance against mechanistic interpretability.

## 4. Discussion and Future Directions

Mouse models have transformed atherosclerosis research, but the next phase of the field will require more precise model selection rather than simply generating more severe disease. *Apoe*^−/−^ and *Ldlr*^−/−^ mice will remain essential because of their robustness, genetic tractability, and extensive historical use. However, the growing availability of humanized lipoprotein models, adult-onset viral induction systems, somatic genome-editing approaches, metabolic syndrome models, and models of plaque complication now allows investigators to align model choice more closely to the biological question.

Studies focused on LDL retention, ApoB100 biology, or familial hypercholesterolemia may be better served by *Ldlr*^−/−^, *Ldlr*^−/−^/*Apobec1*^−/−^, ApoB100-only, or human ApoB transgenic models. Studies of remnant lipoproteins, dysbetalipoproteinemia, CETP biology, or responses to lipid-lowering therapies may benefit from APOE3-Leiden or *APOE*3-Leiden.CETP mice. Immune and inflammatory studies may require ApoE-intact backgrounds, such as *Ldlr*^−/−^ or adult-onset PCSK9-AAV and AAV-CRISPR *Ldlr* approaches, particularly when the pathway of interest intersects with macrophage cholesterol handling or inflammatory regulation. Studies of plaque rupture, coronary disease, or myocardial infarction require specialized models beyond standard *Apoe*^−/−^ and *Ldlr*^−/−^ mice, including *Apoe*^−/−^ Fbn1C1039G^+/−^ and SR-BI-based models.

A second priority is cross-model validation. Many candidate genes and therapeutic interventions have been tested in only one model, most commonly *Apoe*^−/−^ or *Ldlr*^−/−^ mice. Whenever feasible, key mechanistic findings should be validated in a complementary system. For example, an inflammatory pathway identified in *Apoe*^−/−^ mice could be tested in *Ldlr*^−/−^ or PCSK9-AAV models, whereas lipid-modifying interventions may require validation in APOE3-Leiden.CETP mice when the goal is to model human-like lipoprotein-driven atherosclerosis [174] and evaluate lipid-lowering therapies [61]. Discordant results across models should not be viewed merely as failed replication; rather, they may reveal that a pathway is ApoE-dependent, LDL-dependent, remnant-dependent, diet-dependent, sex-dependent, or lesion-stage-specific.

Improved reporting and standardization will also be essential. Atherosclerosis studies are highly sensitive to strain background, sex, age, diet composition, cholesterol and cholate content, feeding duration, housing conditions, microbiome, plasma lipid distribution, lesion site, and quantification method. In viral and somatic editing models, investigators should additionally report vector serotype, dose, route, timing, PCSK9 expression or editing efficiency, hepatic LDLR protein abundance, and criteria for excluding unsuccessful delivery. These variables are not minor technical details; they define the biological context of the model.

Future studies should also move beyond total cholesterol as the main comparator between models. The atherogenicity of a model depends not only on cholesterol concentration but also on the type, size, number, and composition of ApoB-containing particles. *Apoe*^−/−^, *Ldlr*^−/−^, *Ldlr*^−/−^/*Apobec1*^−/−^, APOE3-Leiden, human ApoB, Lp(a), PCSK9-AAV, AAV-CRISPR, and SR-BI-based models each generate distinct lipoprotein landscapes. Lipoprotein fractionation, ApoB48/ApoB100 quantification, HDL measurements, triglycerides, remnant cholesterol, cholesterol efflux assays, CETP activity, and Lp(a) measurements should therefore be incorporated whenever relevant.

Adult-onset induction systems represent an important opportunity. PCSK9-AAV and AAV-CRISPR *Ldlr* approaches allow atherosclerosis to be induced in existing floxed, Cre-driver, reporter, aged, or disease-prone mice without years of breeding. These models are particularly useful for studying aging, clonal hematopoiesis, obesity, diabetes, chronic inflammation, and complex immune pathways. However, they are not interchangeable with germline *Ldlr*^−/−^ mice. PCSK9-AAV produces supraphysiological PCSK9 expression, whereas AAV-CRISPR depends on editing efficiency, AAV tropism, sex, strain, and age at delivery. Future work should define the contexts in which these approaches faithfully reproduce classical models and those in which they introduce distinct biology.

Better modelling of immunometabolic disease is another major need. Human atherosclerosis usually develops in the setting of multiple interacting risk factors, including obesity, insulin resistance, aging, hypertension, chronic inflammation, and altered hematopoiesis. Combining atherosclerosis-prone backgrounds or adult-onset PCSK9-AAV induction with metabolic stress, aging, or inflammatory challenges may better capture residual inflammatory risk. These models are more clinically relevant but less mechanistically simple, and experiments should therefore be designed to distinguish the contributions of lipids, glucose metabolism, adipose inflammation, hematopoietic remodelling, and vascular inflammation.

The field should also continue improving models of advanced plaque complications. Standard *Apoe*^−/−^ and *Ldlr*^−/−^ mice are excellent for studying lesion initiation and progression, but they rarely reproduce spontaneous plaque rupture, coronary thrombosis, myocardial infarction, or sudden death. Models such as *Apoe*^−/−^ Fbn1C1039G^+/−^, SR-BI^−/−^/*Apoe*^−/−^, SR-BI^−/−^/*Ldlr*^−/−^, and SR-BI^−/−^/ApoeR61^h/h^ mice may be more appropriate for studying plaque vulnerability, coronary disease, and clinical-event-like phenotypes. These specialized models should complement, not replace, classical models.

Finally, future studies should place greater emphasis on plaque composition, spatial biology, and computational benchmarking against human disease. Lesion area remains informative, but plaque size alone does not capture necrotic-core formation, fibrous-cap structure, smooth-muscle-cell plasticity, macrophage states, calcification, intraplaque haemorrhage, efferocytosis, adventitial inflammation, or immune-cell organization. AI-assisted pathology can automate and standardize the segmentation and quantification of lesion burden, plaque distribution, and composition across large histological and three-dimensional imaging datasets [175,176]. Spatial transcriptomics, spatial proteomics, multiplex imaging, and lineage tracing can further connect molecular cell states with defined plaque compartments and reveal spatially restricted cellular interactions that are lost during tissue dissociation [177,178]. Integrating these approaches with single-cell datasets, lipoprotein profiles, immune phenotyping, histopathology, and therapeutic responses through machine-learning methods could enable multidimensional comparisons among mouse models and human disease, helping investigators identify which model best reproduces a specific biological or pathological feature. However, these tools will require representative training datasets, external validation across strains, sexes, diets, vascular sites, and imaging platforms, transparent reporting, and continued expert oversight to prevent technical or algorithmic differences from being misinterpreted as biological variation.

### Sex as a Biological Variable in Model Selection and Interpretation

Sex substantially influences experimental atherosclerosis, although the magnitude and direction of these effects depend on the model, age, diet, vascular site, and analytical endpoint [179]. In young *Apoe*^−/−^ mice, females often develop larger aortic-root lesions than males, whereas this difference diminishes with age [179,180]. In *Ldlr*^−/−^ mice, females may also develop larger aortic-root lesions, while males can show greater disease when the entire aorta is assessed [179]. Sex-dependent phenotypes should therefore not be generalized across vascular sites or experimental designs.

The greater lesion burden observed in young females does not imply that estrogen is proatherogenic. Ovariectomy increases lesion formation in *Apoe*^−/−^ mice, whereas 17β-estradiol replacement reduces atherosclerosis [181]. Similar protection is observed in *Ldlr*^−/−^ mice, even without major changes in plasma cholesterol, supporting direct vascular and immunoregulatory effects of estrogen [182]. Thus, sex, gonadal status, hormone levels, and timing of hormone exposure should be considered distinct experimental variables [179].

Sex may also affect plaque composition independently of lesion size. Male mice can develop smaller but more inflammatory lesions, whereas females may exhibit greater plaque burden with distinct macrophage, inflammasome, and adaptive immune responses [179]. Genetic or pharmacological interventions may therefore produce different outcomes in males and females. Studies should assess lesion composition and inflammatory endpoints in addition to plaque area and plasma cholesterol and should initially analyze each sex separately.

AAV-PCSK9 models have been validated in female mice, but their efficiency can be sex dependent. At the same low vector dose, AAV8-mediated mutant PCSK9 expression can produce lower circulating PCSK9 and cholesterol levels in females than in males, although higher doses induce hypercholesterolemia in both sexes [183]. Vector performance also depends on the strategy used to reduce LDLR. In direct comparisons, AAV-hPCSK9 produced stronger atherosclerosis in females, whereas AAV-CRISPR-mediated *Ldlr* disruption was more effective in males [55].

These findings have practical implications for study design. AAV-based studies should report sex, age, vector serotype and dose, construct, delivery route, diet, circulating PCSK9, hepatic LDLR abundance, plasma lipoproteins, and lesion site. Pilot studies should confirm comparable induction of hypercholesterolemia in each sex, and therapeutic effects should be evaluated independently because sex-dependent differences in lesion burden, immune activation, hormone status, and vector efficiency may alter both treatment magnitude and mechanism [55,179,183].

In summary, the future of mouse atherosclerosis research will depend on using models more strategically. No model is universally superior; each has distinct strengths, limitations, and translational applications. The goal should be to match the model to the biological question, report the experimental context rigorously, and interpret findings within the disease axis that each model is designed to interrogate.

## Figures and Tables

**Table 1:** Comparative characteristics and recommended applications of mouse models of atherosclerosis. This table provides a practical comparison of commonly used and specialized mouse models of atherosclerosis. Models are organized according to the principal disease feature they are designed to interrogate, including lipoprotein-driven lesion development, immunoinflammatory and immunometabolic mechanisms, adult-onset hypercholesterolemia, humanized lipoprotein biology, plaque complexity, coronary artery disease, and myocardial infarction-like outcomes. For each model, the table summarizes the underlying genetic or experimental manipulation, predominant lipoprotein abnormality, dietary dependence, characteristic lesion phenotype, major strengths, principal limitations, and research questions for which the model is best suited. The categories are intended as practical guidance rather than rigid classifications, because several models can be applied across multiple areas of atherosclerosis research. Model performance may also vary according to genetic background, sex, age, diet composition, duration of feeding, microbiota, vascular site, and experimental endpoint.

Disease Axis/Primary Use	Model	How Disease Is Induced	Main Lipid/Biological Context	Typical Lesion/Phenotype	Best Used for	Major Interpretation Hazard	Key Source(s)
1. Lipoprotein-driven lesion initiation and progression	Apoe−/−	Germline ApoE deletion; chow or Western-type diet	Remnant-rich hypercholesterolemia; accumulation of VLDL/chylomicron remnants; complete loss of ApoE	Robust spontaneous aortic root and aortic lesions; accelerated by Western diet	Rapid, reproducible lesion formation; broad mechanistic studies; historical comparison; inflammation and immune-cell recruitment studies	ApoE has roles beyond lipid clearance, including macrophage cholesterol handling, reverse cholesterol transport, inflammation, and immune regulation	PMID: 27386935/PMID: 31032262/PMID: 38428154
	Ldlr−/−	Germline LDL receptor deletion; usually requires Western-type or cholesterol-rich diet	LDL/ApoB-driven hypercholesterolemia with preserved ApoE	Diet-dependent aortic root, aortic arch, brachiocephalic, and en face aortic lesions	LDL-driven atherogenesis; diet modulation; candidate genes; immune studies with ApoE intact	Strongly diet-dependent; outcomes sensitive to diet composition, sex, microbiota, strain, and feeding duration	PMID: 27386935/PMID: 37174655/PMID: 38428154
	Apoe−/−/Ldlr−/−	Combined ApoE and LDLR deletion; often with high-fat/high-cholesterol diet	Severe remnant and LDL/VLDL accumulation	More severe and advanced lesions than single knockouts; possible coronary involvement with prolonged diets	Severe hyperlipidemic disease burden; accelerated lesion studies	Very severe and artificial lipid phenotype; loss of both major clearance pathways limits physiological interpretation	PMID: 28483459/PMID: 33258000
	Ldlr−/−/Apobec1−/−	LDLR deletion plus loss of ApoB mRNA editing	High ApoB100 and LDL-C; more human familial hypercholesterolemia-like	Extensive atherosclerosis even on chow or low-fat diet; lipid core and fibrous cap features with age	LDL-C/ApoB100-driven disease; familial hypercholesterolemia; LDL retention biology	Less commonly used; more complex breeding; less practical for large genetic studies	PMID: 9701246/PMID: 29977908
	ApoB100-only/Ldlr−/− ApoB100 models	Genetic manipulation to favor ApoB100-containing particles	Human-like ApoB100-rich LDL context	LDL-driven lesion development depending on background and diet	ApoB100 particle biology; LDL retention; lipoprotein size/number questions	Not a general workhorse model; specialized for ApoB100/LDL biology	PMID: 27386935/PMID: 9701246
	Human ApoB transgenic mice	Transgenic expression of human ApoB	Human ApoB100 largely in LDL-sized particles; increased ApoB-containing lipoproteins	Severe aortic lesions after high-fat/high-cholesterol diet; limited disease on chow	Human ApoB production; LDL-like particle biology; diet-induced ApoB-driven disease	Requires dietary challenge; less convenient and less widely used than Apoe−/− or Ldlr−/−	PMID: 7738190
	Human ApoB + apo(a)/Lp(a) transgenic mice	Human ApoB transgene crossed with human apo(a) transgene	Formation of human-like Lp(a), which mice do not naturally produce	Specialized lipoprotein phenotype; atherosclerosis context depends on diet/background	Lp(a) biology; apoB100–apo(a) interaction; humanized lipoprotein risk	Highly specialized model; not a broad model of plaque progression	PMID: 8254057
	APOE*3-Leiden	Human APOE*3-Leiden transgene; diet-responsive	Familial dysbetalipoproteinemia-like remnant lipoprotein metabolism; endogenous mouse ApoE preserved in macrophages	Diet-induced lesions from fatty streaks to advanced plaques with foam cells, oxidized LDL epitopes, fibrosis, and necrotic features	Remnant metabolism; diet responsiveness; dysbetalipoproteinemia-like disease	Slower and more diet-dependent than Apoe−/−; transgene expression and diet composition matter	PMID: 7683682/PMID: 8163645/PMID: 9544741
	APOE*3-Leiden.CETP	APOE*3-Leiden crossed with human CETP transgene	Humanized lipid distribution; CETP shifts cholesterol from HDL toward VLDL/LDL	Increased VLDL/LDL cholesterol, reduced HDL, reduced SR-BI-mediated cholesterol efflux, increased lesion area	Human-like lipoprotein metabolism; CETP biology; lipid-lowering therapy studies	Less genetically flexible; more complex and diet-dependent; not ideal for rapid mechanistic crosses	PMID: 7683682/PMID: 38428154
1. Lipoprotein-driven initiation/2. Immunometabolic mechanisms	PCSK9-AAV	AAV-mediated hepatic expression of gain-of-function PCSK9	Adult-onset LDLR degradation; rapid hypercholesterolemia	Aortic atherosclerosis after AAV injection plus Western/high-fat diet	Rapid induction in existing strains; conditional alleles; reporter mice; aging studies; complex genetic backgrounds	Supraphysiological PCSK9 expression; possible PCSK9-specific effects beyond LDLR degradation; AAV/vector effects	PMID: 24677271/PMID: 25341796/PMID: 30026278
	AAV-CRISPR Ldlr	AAV-delivered Cas9/gRNA targeting hepatic Ldlr	Adult-onset somatic Ldlr disruption	Severe hypercholesterolemia and atherosclerosis after Western diet	Rapid adult-onset genetic LDLR loss; candidate gene testing; genetic interaction studies	Editing efficiency, off-target effects, vector controls, and sex differences must be considered	PMID: 28300165/PMID: 30026278
2. Immunoinflammatory and immunometabolic mechanism models	Ldlr−/− as immune-study backbone	LDLR deletion plus controlled diet	Hypercholesterolemia with ApoE intact	Diet-dependent lesions with immune-cell recruitment and foam cell formation	Macrophages, monocytes, T cells, cytokines, inflammasome, trained immunity, bone marrow chimeras, conditional alleles	Diet can dominate immune phenotype; requires careful matching of diet, sex, age, and plasma lipids	PMID: 31032262/PMID: 27386935
	Apoe−/− as immune-study backbone	ApoE deletion; chow or Western diet	Strong inflammatory/remnant-rich atherosclerotic context with ApoE absent	Robust lesions; extensive literature for immune pathways	Historical comparison; strong lesion burden; inflammatory pathway screening	Immune effects may reflect ApoE deficiency rather than general atherosclerosis biology	PMID: 27386935/PMID: 19302038/PMID: 34389841
	PCSK9-AAV in floxed/reporter/aged mice	AAV-PCSK9 injection into existing genetic backgrounds	Adult-onset hypercholesterolemia without breeding to Apoe−/− or Ldlr−/−	Aortic lesions after diet; compatible with complex immune genetics	Cell-specific genes, reporters, aging, clonal hematopoiesis, sterile inflammation, immunometabolism	Vector exposure and PCSK9 biology can confound immune interpretation	PMID: 30026278/PMID: 31032262
	AAV-PCSK9 + HFD-C	AAV-PCSK9 plus high-fat diet with added cholesterol	Combined hypercholesterolemia, obesity/metabolic stress, insulin resistance, adipose inflammation	Atherosclerosis plus impaired glucose clearance, insulin resistance, pancreatic islet hyperplasia, adipose Ly6Chigh/med monocyte expansion	Atherosclerosis with metabolic syndrome; obesity-associated inflammation; systemic immunometabolism	Diet effects may dominate phenotype; difficult to separate lipids, glucose, adiposity, and inflammation	PMID: 35919346
	Ldlr−/− or Apoe−/− + ob/ob or db/db	Atherosclerosis-prone background crossed with obesity/diabetes mutations	Hyperlipidemia plus obesity, insulin resistance, or diabetes	Accelerated lesions with systemic metabolic inflammation	Diabetes/obesity-accelerated atherosclerosis; adipose-vascular immune crosstalk	Severe metabolic disturbance; difficult to distinguish direct vascular effects from systemic metabolism	PMID: 31032262/PMID: 28483459
	Apoe−/− or Ldlr−/− + aging/chronic inflammatory triggers	Atherosclerosis background plus age or inflammatory exposure	Hyperlipidemia plus altered immune tone, inflammaging, or systemic inflammatory priming	Context-dependent lesion acceleration and immune remodeling	Aging, sterile inflammation, trained immunity, systemic inflammatory risk	Aging and chronic inflammation alter many systems simultaneously; requires careful controls	PMID: 34389841/PMID: 31032262
3. Plaque complexity, rupture, coronary disease, and clinical-event-like phenotypes	Apoe−/− Fbn1C1039G+/−	ApoE deletion plus fibrillin-1 mutation; Western diet	Hyperlipidemia plus altered extracellular matrix integrity and arterial stiffening	Large vulnerable plaques; plaque rupture-like events; intraplaque hemorrhage; atherothrombosis; MI/stroke-like outcomes in some reports	Plaque vulnerability, matrix remodeling, atherothrombosis, plaque-stabilizing therapies	Fibrillin mutation creates nonstandard vessel-wall biology; not a general model of atherogenesis	PMID: 28483459/PMID: 31032262
	Apoe−/− + Angiotensin II	ApoE deletion plus high-fat diet and AngII infusion	Hyperlipidemia plus hypertension, vascular inflammation, oxidative stress, aneurysm-prone biology	Plaque destabilization, vulnerable plaque features, aneurysm formation, rupture-prone phenotype	Vascular stress, inflammation, hypertension-associated destabilization	AngII has broad systemic effects; not a clean spontaneous plaque rupture model	PMID: 28483459/PMID: 31032262
	SR-BI−/−/Apoe−/−	Combined SR-BI and ApoE deletion	Severe dyslipidemia, abnormal HDL/unesterified cholesterol-rich lipoproteins, impaired reverse cholesterol transport	Occlusive coronary lesions, spontaneous MI, cardiac dysfunction, ECG abnormalities, premature death	Coronary artery disease, MI-like complications, cardiac dysfunction	Very severe early phenotype; abnormal lipoprotein particles; limited for slow-progressing disease	PMID: 11861414/PMID: 9356497/PMID: 33258000
	SR-BI−/−/ApoeR61h/h	SR-BI deletion plus hypomorphic apoE; atherogenic diet	Diet-inducible coronary heart disease-like phenotype	Occlusive coronary atherosclerosis, MI, cardiac dysfunction, premature death after atherogenic diet	Diet-controlled onset of coronary disease; intervention and regression studies	Specialized SR-BI/apoE biology; not a standard atherosclerosis model	PMID: 16200214/PMID:33258000
	SR-BI−/−/Ldlr−/−	Combined SR-BI and LDLR deletion; atherogenic diet	LDLR loss plus disturbed HDL/SR-BI pathway	Coronary lesions and myocardial pathology in severe settings	Coronary atherosclerosis with ApoE preserved; ischemia/cardioprotection context	Complex lipid phenotype with altered HDL, LDLR, and reverse cholesterol transport	PMID: 33258000/PMID: 9356497
	Apoe−/−/Ldlr−/− with prolonged diet	Dual deletion plus prolonged high-fat/high-cholesterol diet	Severe remnant and LDL/VLDL accumulation	Advanced lesions; possible coronary involvement with long protocols	Severe disease burden; advanced lesion development	Does not consistently reproduce spontaneous rupture or MI; severe artificial lipid context	PMID: 33258000/PMID: 28483459
4. Cross-cutting practical variable	Diet composition	Chow, Western diet, high-fat/high-cholesterol diet, cholate-containing diet, HFD-C	Determines lipid profile, inflammation, metabolic stress, and lesion kinetics	Can shift early lesions to advanced disease or induce metabolic syndrome	Tuning disease severity and biological context	Diet differences can make studies difficult to compare	PMID: 38428154/PMID: 33977048/PMID: 35919346
	Genetic background	C57BL/6, C3H/HeJ, FVB, 129, mixed backgrounds	Modifies lipid metabolism, immune tone, vascular susceptibility	Can alter lesion size, lesion site, and response to intervention	Susceptibility studies; genetic modifiers	Findings may not generalize across backgrounds	PMID: 37174655/PMID: 27386935
	Endpoint/lesion site	Aortic root, en face aorta, brachiocephalic artery, carotid, coronary artery	Different sites may be driven by different lipid, flow, and immune mechanisms	Lesion burden and composition can differ by site	Matching endpoint to biological question	Aortic root lesion area is not equivalent to plaque rupture, coronary disease, or clinical events	PMID: 38428154/PMID: 33258000

**Table 2:** Representative experimental timelines and lesion stages in commonly used mouse models of atherosclerosis. This table provides practical guidance on the approximate time required to generate early and more complex atherosclerotic lesions in commonly used experimental models, including chow-fed *Apoe*^−/−^ mice, Western diet-fed *Ldlr*^−/−^ mice, and AAV-PCSK9-induced models. For each model, the table summarizes the dietary or experimental induction strategy, representative windows for early and advanced lesion development, typical research applications, and major factors that may influence disease progression. These timelines are intended as practical starting points rather than fixed thresholds, because lesion development varies according to genetic background, sex, age at study initiation, diet composition, feeding duration, vascular site, vector serotype and dose, efficiency of LDLR depletion, microbiota, and the criteria used to define lesion stage. Investigators should therefore verify plasma lipoprotein levels and plaque characteristics within their specific experimental setting.

Model	Diet or Induction Strategy	Representative Early-Lesion Window	Representative Complex-Lesion Window	Typical Applications	Important Considerations
*Apoe*−/−	Standard chow	Approximately 12–16 weeks of age	Approximately 24–40 weeks of age	Spontaneous lesion initiation and progression without dietary induction	Lesion kinetics vary with sex, genetic background, vascular site, and housing conditions. Western diet substantially accelerates progression.
*Ldlr*−/−	Western-type diet	Approximately 8–12 weeks of feeding	Approximately 16–24 weeks of feeding	Diet-controlled LDL/ApoB-driven lesion initiation, progression, and plaque-composition studies	Outcomes are highly sensitive to dietary cholesterol, fat source, cholate content, sex, microbiome, and starting age.
PCSK9-AAV	AAV-mediated expression of gain-of-function PCSK9 followed by Western-type or high-fat/high-cholesterol diet	Lesions are commonly evaluated within 8–16 weeks after injection	More complex lesions generally require the longer portion of this interval or extended feeding	Rapid, adult-onset induction of hypercholesterolemia in existing genetic, reporter, aged, or disease-prone strains	Kinetics depend on AAV serotype, vector dose, PCSK9 construct, sex, strain, hepatic transduction, diet, and degree of LDLR depletion.

## Data Availability

No new data were created or analyzed in this study. Data sharing is not applicable to this article.

## Abbreviations

The following abbreviations are used in this manuscript:

AAV	Adeno-associated virus
AngII	Angiotensin II
apo(a)	Apolipoprotein(a)
ApoB	Apolipoprotein B
ApoB48	Apolipoprotein B48
ApoB100	Apolipoprotein B100
ApoE	Apolipoprotein E
CCL2	C-C motif chemokine ligand 2
CCR2	C-C motif chemokine receptor 2
CD4	Cluster of differentiation 4
CETP	Cholesteryl ester transfer protein
CRISPR	Clustered regularly interspaced short palindromic repeats
CX3CL1	C-X3-C motif chemokine ligand 1
CX3CR1	C-X3-C motif chemokine receptor 1
HDL	High-density lipoprotein
HFD-C	High-fat, high-cholesterol diet
ICAM-1	Intercellular adhesion molecule 1
IFN	Interferon
LAD	Left anterior descending
LDL	Low-density lipoprotein
LDL-C	Low-density lipoprotein cholesterol
LDLR	Low-density lipoprotein receptor
Lp(a)	Lipoprotein(a)
MacAIR	Aortic intima-resident macrophage(s)
MCP-1	Monocyte chemoattractant protein 1
MI	Myocardial infarction
PCSK9	Proprotein convertase subtilisin/kexin type 9
RNA-seq	RNA sequencing
scRNA-seq	Single-cell RNA sequencing
SR-BI	Scavenger receptor class B type I
Th1	T helper type 1
TREM2	Triggering receptor expressed on myeloid cells 2
VCAM-1	Vascular cell adhesion molecule 1
VLDL	Very-low-density lipoprotein
